# Numerical Calculation of Energy Performance and Transient Characteristics of Centrifugal Pump under Gas-Liquid Two-Phase Condition

**DOI:** 10.3390/mi11080728

**Published:** 2020-07-28

**Authors:** Ling Zhou, Yong Han, Wanning Lv, Yang Yang, Yong Zhu, Xiangyu Song

**Affiliations:** 1 National Research Center of Pumps, Jiangsu University, Zhenjiang 212013, China; lingzhou@ujs.edu.cn (L.Z.); 2211811008@stamail.ujs.edu.cn (Y.H.); 2211911018@stamail.ujs.edu.cn (W.L.); yangyang_ujs@outlook.com (Y.Y.); 2 Department of Mechanical, Engineering of Texas Tech University, Lubbock, TX 79411, USA; 3 Zhejiang Zhenxing Petrochemical Machinery Co. Ltd., Wenzhou 325204, China; sxy@zhenxingcn.cn

**Keywords:** gas-liquid two-phase flow, centrifugal pump, transient characteristics, numerical calculation, test verification

## Abstract

The unstable operation of a centrifugal pump under the gas-liquid two-phase condition seriously affects its performance and reliability. In order to study the gas phase distribution and the unsteady force in an impeller, based on the Euler-Euler heterogeneous flow model, the steady and unsteady numerical calculations of the gas-liquid two-phase full flow field in a centrifugal pump was carried out, and the simulation results were compared with the test data. The results show that the test results are in good agreement with the simulation data, which proves the accuracy of the numerical calculation method. The energy performance curve of the model pump decreases with the increase of the gas content, which illustrates a serious impact on the performance under the part-load operating condition. The results reveal that the high-efficiency-operating range become narrow, as the gas content increases. The gas phase is mainly distributed on the suction surface of the impeller blades. When the gas content reaches a certain value, the gas phase separation occurs. As the inlet gas content increases, the radial force on the impeller blades decreases. The pattern of the pressure pulsation is similar to that under pure water and low gas content conditions, and the number of peaks during the pulsation is equal to the number of the impeller blades. After the gas content reaches a certain value, the pressure fluctuates disorderly and the magnitude and the direction of radial force change frequently, which are detrimental to the operation stability of the pump. The intensity of the pressure pulsations in the impeller flow channel continues to increase in the direction of the flow under pure water conditions. The pressure pulsation intensities at the blade inlet and the volute tongue become more severe with the increase of the gas content.

## 1. Introduction

Centrifugal pumps are extensively employed in engineering fields, and they play an indispensable role in the daily life [1]. The shortage of energy resources has put forward requirements for the higher performance of centrifugal pumps and the improvement of the stability in the pump operation, which has a direct effect on the reduction of energy consumption [2,3]. In engineering applications, inevitably, centrifugal pumps mix multiphase substances in a pumping medium, which has a certain impact on the characteristics of the pump [4,5]. In serious cases, the impeller may even become blocked, resulting in severe consequences and accidents [6,7].

It is of great practical value to study the distribution and the mechanism of a gas-liquid two-phase flow in centrifugal pumps, which is especially crucial in the fields of petroleum exploitation, natural gas transportation, and chemical industry [8,9,10]. He et al. [11] and Ge et al. [12] proposed the numerical simulation method of Computational Fluid Dynamics (CFD) and Population Balance Modeling (PBM) coupling to numerically calculate a gas-liquid two-phase flow on a centrifugal pump, and the numerical simulation results matched the experimental results well, which led to the conclusion that the method is superior to the Eulerian–Eulerian model. Sato et al. [13] confirmed through performance testing and visual experiments that making adjustments to the geometry of the impeller, such as drilling the backflow holes, increasing the blade outlet placement angle, and opening the impeller, can improve the performance of the pump under the conditions of a gas-liquid two-phase flow. In order to verify the accuracy of the common gas-liquid two-phase-flow numerical model and the effect of installing an inducer or increasing the tip clearance on the performance of a centrifugal pump, Trupen et al. [14] adopted the volume of fluid (VOF) method and the Shear-Stress Transport (SST) k-ω turbulence model to numerically calculate the centrifugal pump. Poullikkas [15] studied the pump head under gas-liquid two-phase flow conditions with the theory of the controlled volume model, which combines the multiscale effects of pump structure parameters, phase separation, density change, and gas phase compressibility. The results showed that the interaction between the gas phase and the liquid phase in the impeller directly affected the performance of the pump. Matsushita et al. [16] utilized performance testing and high-speed photography techniques to study the application of the similarity law to the back-bent centrifugal pump and found that the impeller diameter and the rotation speed still followed the similarity law under gas-liquid two-phase flow conditions. Pirouzpanah et al. [17] used electrical resistance tomography (ERT) to measure the internal flow of electric submersible pumps. The experimental results showed that the gas distribution patterns have a direct impact on pump performance. Caridad et al. [18] performed numerical calculations to study the performance of an electric submersible centrifugal pump. The effects of the relative liquid flow angle of the outlet and the flow rate under gas-liquid two-phase conditions were analyzed. Finally, the accuracy of the numerical calculation was verified by experiments. Verde et al. [19] used high-speed photography to test the internal flow pattern of a two-phase-flow centrifugal pump. They found that there are four typical flow patterns in the impeller: bubbly flow, polymerization bubbly flow, air mass flow, and slug flow. Cubas et al. [20] conducted a performance test of a two-pole centrifugal pump with radial-type impellers and a vaned diffuser and conducted a visual experimental study on the distribution of air bubbles inside the first-stage impeller and diffuser. Kosmowski et al. [21] found that the pressure pulsation in the impeller flow channel was the main source for the gas-liquid separation and the accumulation of the gas phase caused a sharp drop in the pump performance. When the gas content was higher than 15%, the phase separation occurred at the outlet of the impeller. Once the gas content exceeded 20%, the flow interruption might occur. Under the conditions of different inlet gas contents, the research on the radial force distribution of the centrifugal pump and the transient pressure change in the impeller flow channel is still lacking.

The research purpose of this paper is a single-stage single-suction volute centrifugal pump with a specific speed n_s_ of 85. Based on the Euler-Euler heterogeneous flow model, the steady numerical calculation of the centrifugal pump was carried out under different flow conditions, and the accuracy of the numerical calculation was verified by comparing the predicted performance with the test results. Unsteady simulations were implemented to obtain the distribution of the gas phase on the cross-section of the centrifugal pump impeller under different inlet gas contents, and the pressure pulsations and the radial forces at different locations of the pump were analyzed.

## 2. Geometric Model and Numerical Methods

### 2.1. Geometric Model

The model pump [22] was a single-stage single-suction volute centrifugal pump with a specific speed n_s_ of 85. The main design parameters of the centrifugal pump are shown in Table 1.

**Figure 1:**
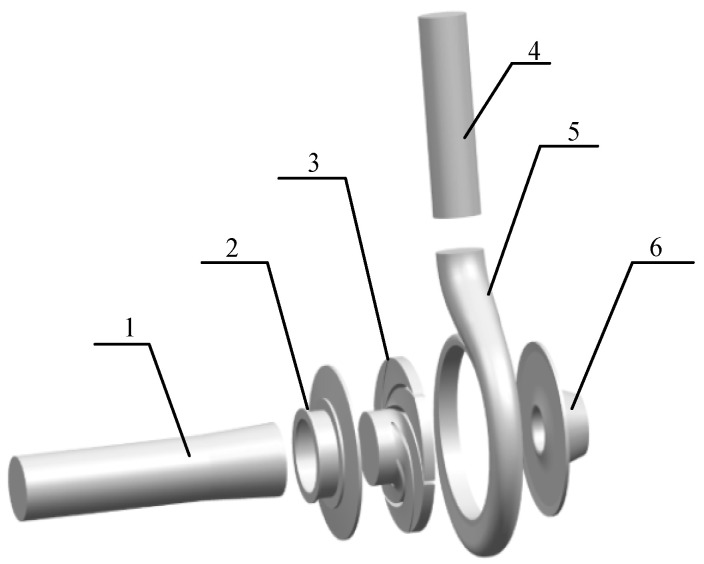
Pump model used in the simulation: **1.** inlet pipe; **2.** front chamber; **3.** impeller; **4.** outlet pipe; **5.** volute; **6.** rear chamber.

The three-dimensional software UG10.0 was used to model the three-dimensional full flow field of the model pump, and the clearance gap was included to consider the effects of the leakage on the performance of the pump, as shown in Figure 1. The calculation domain included an inlet pipe, a pump chamber, an impeller, a volute, and an outlet pipe. The pump chamber included a front chamber and a rear chamber. In order to achieve fully developed flow conditions, the inlet and outlet pipes of the calculation domain were appropriately extended.

### 2.2. Meshing

**Figure 2:**
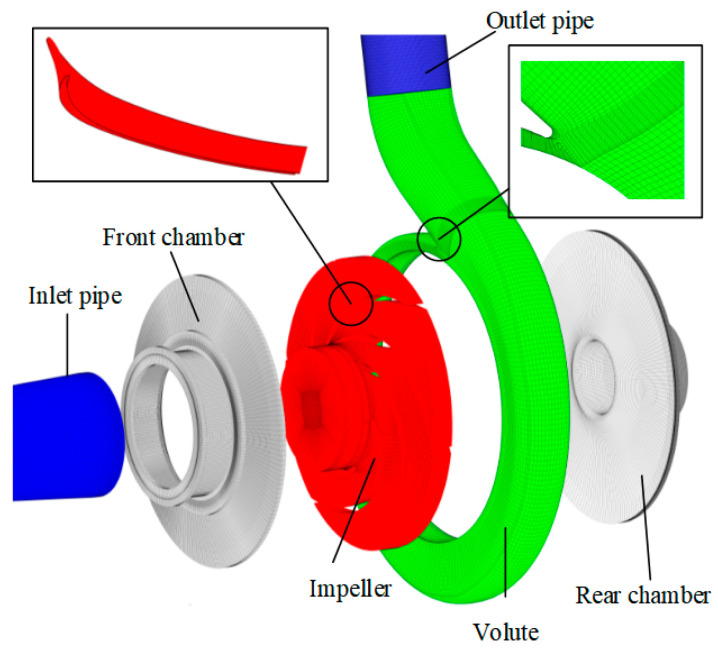
Computational domain and structured grid.

Compared with an unstructured grid, a structured grid has an orderly arrangement of nodes and adjacent points, which can effectively adjust the nodes on each layer of the grid line [23]. To obtain the high-qualified grid and ensure the accuracy of numerical calculation [24,25], this article applied the ANSYS-ICEM 17.0 software to create the hexahedral structured mesh for each part of the calculation domain. The mesh near the wall was refined by the boundary layer to meet the requirement of wall functions [26,27,28]. The overall structured grid of the calculation domain is shown in Figure 2.

**Figure 3:**
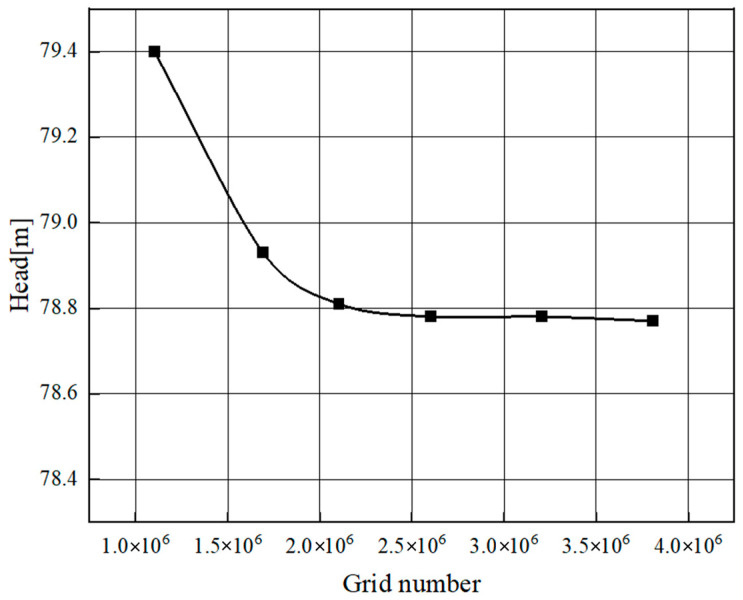
Grid independence analysis.

Figure 3 shows the numerical simulation results under pure water conditions with different grid numbers. Comparing the efficiency, head, and power, we could find that the numerical results were getting stable with the increase of grid number. By considering the calculation cost and accuracy, the scheme with a total number of grids of 2,104,583 was finally selected for the following numerical calculation.

### 2.3. Boundary Conditions

In this paper, the ANSYS-CFX17.0 software was selected for the numerical calculation based on the Euler-Euler heterogeneous flow model. The liquid-phase turbulence model used the SST k-ε turbulence model, and the gas-phase turbulence model used the zero-equation model. In the steady calculation, the inlet boundary condition was set to the total pressure inlet, and the outlet boundary condition was set to the mass flow outlet. The dynamic and static interface contact method between the impeller and the volute was in the Frozen Rotor mode, and the wall surface was an adiabatic and nonslip wall surface. In the unsteady calculation, the steady calculation result was used as the initial condition of the unsteady calculation for transient simulation, and the contact mode of the dynamic and static interface was set as the Transient Rotor Stator mode. The impeller was rotated by 3° for each time step, i.e., every 0.175 × 10^−4^ s in the calculation. Accordingly, one rotation period had 120 steps in total. The total number of rotation periods was 8, and the total calculation time was 0.1684 s.

## 3. Results and Discussion

### 3.1. Test Validation

**Figure 4:**
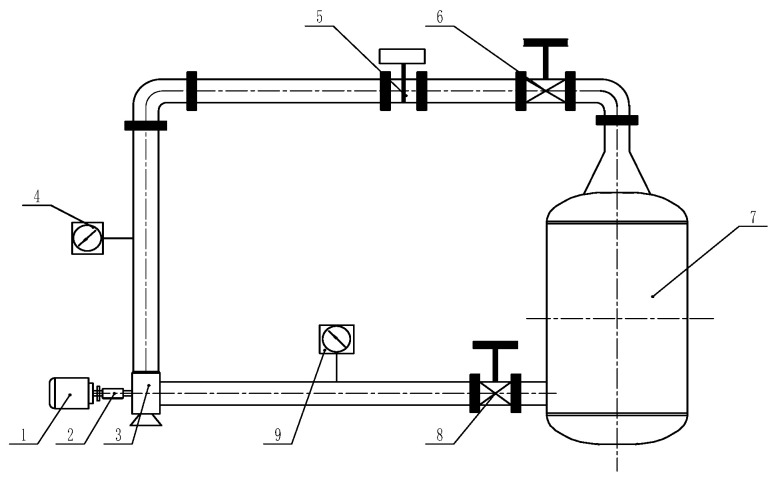
Schematic of test rig: **1.** motor; **2.** torque meter; **3.** centrifugal pump; **4.** outlet pipeline pressure gauge; **5.** DN100 electromagnetic flowmeter; **6.** outlet pipeline valve; **7.** water tank; **8.** inlet pipeline valve; **9.** inlet pipeline pressure gauge.

**Figure 5:**
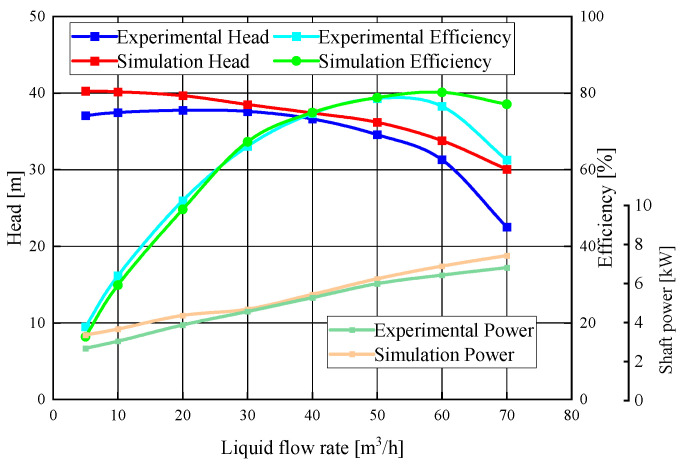
Comparison of the experimental test and the numerical results.

In order to verify the accuracy of the numerical calculation, the test bench shown in Figure 4 was established to carry out the energy performance test under different working conditions. A DN100 electromagnetic flowmeter with an accuracy of 0.5% was used to measure the flow rate. A WT2000 intelligent differential pressure transmitter and a torque meter with accuracies of 0.1% and 0.2%, respectively, were used to measure the head and torque, respectively. Figure 5 shows the comparison between the numerical results and the experiment data of the pump head, efficiency, and power. In order to fully analyze the changing trend of energy performance from the shut-off flow rate to the maximum flow rate, a total of eight operating flow rates were selected in the numerical simulation. The relative error of each curve tended to increase in the region deviating from the design flow rate (e.g., 5 and 70 m^3^/h), which was due to the phenomena of the severe turbulent flow and back-flow of the centrifugal pump operating in this region, resulting in an increase in the error between the numerical simulation and the experimental results. However, the tendency of each corresponding curve was almost the same. Under the design flow rates, the relative errors of the head, the efficiency, and the power between the numerical calculation and the experimental test were 4.4%, 2.95%, and 4%, respectively. The relative errors were all less than 5%, which indicated that the numerical simulation method chosen in this study is relatively precise.

### 3.2. Influence of the Gas Content on Energy Performance

**Figure 6:**
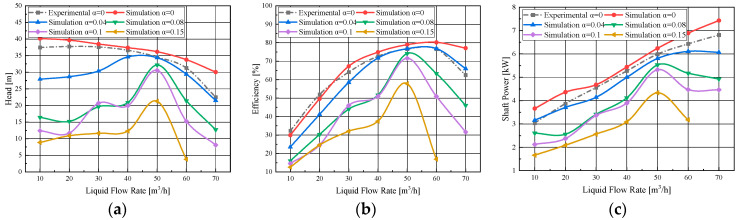
Effect of the inlet gas fraction on the energy performance of the pump: (**a**) flow–head curve; (**b**) flow–efficiency curve; (**c**) flow–shaft power curve.

Figure 6 shows the comparison of head, efficiency, and shaft power under the condition of different inlet gas contents at a rated speed of 2850 rpm. It can be seen from Figure 6a that the head under different gas-liquid two-phase flow conditions was always lower than that under pure water flow conditions. Under different inlet gas content conditions, the head reached the maximum value at the design flow point, which was also different with the pure water condition. The inlet gas content had a dramatic effect on the performance of the pump under nondesign flow conditions. Figure 6b,c shows the efficiency and shaft power under different inlet gas content conditions. The curve of each parameter increased with flow, and it is similar to that of the flow-head curve. With the growth of inlet gas content, the high efficiency range of the model pump was reduced, and the efficiency under over-load flow rates decreased rapidly. The reason may be that the flow pattern in the impeller channels was changed under the gas-liquid two-phase flow condition [29]. After the gas content reached a certain value, gas blocking appeared in the impeller channels [20]. The transfer of the mechanical energy to the kinetic energy of the liquid during the impeller rotation was blocked, resulting in the reduction of head and efficiency.

### 3.3. Effect of the Inlet Gas Content on the Gas Distribution

**Figure 7:**
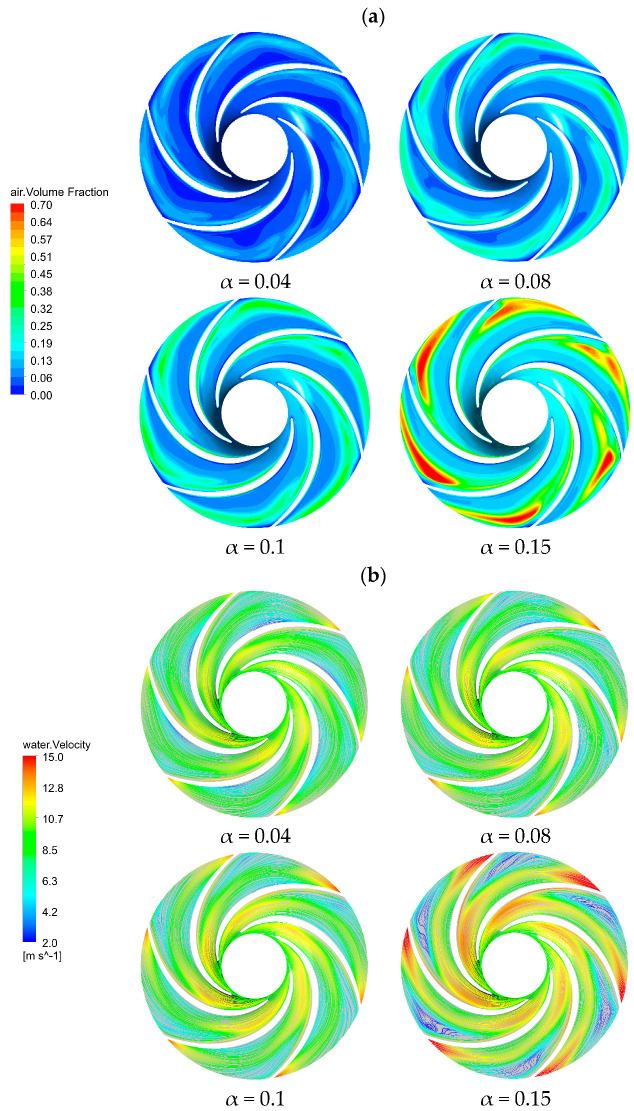
The cross-section of the impeller: (**a**) gas distributions under different inlet gas fractions, (**b**) liquid-phase velocity streamlines distribution under different inlet gas fractions.

Different inlet gas contents have different effects on the performance of a centrifugal pump. With the increase of gas content, the proportion of the gas phase distribution in the impeller flow channel is enhanced, which hinders the normal delivery of the liquid phase. Once the gas phase fraction reaches a certain value, the gas lock phenomenon occurs in the impeller channels, which has a serious impact on the pump’s operating stability. Figure 7a shows the gas distribution on the cross-section of the impeller under four different inlet gas content conditions. The gas phase was mainly distributed on the suction surface of the impeller blades and was gradually gathered along the impeller flow path toward the outlet.

As the inlet gas content increased, the proportion of gas content increased, while the space of liquid phase was squeezed. The gas was gradually extended from the suction surface of the blades to the pressure surface, but the gas was still mainly distributed near the suction surface and the outlet of the blades, due to the disparity in the density and the mass of the gas-liquid two-phase flow. Compared with the gas phase, the liquid phase had higher density and mass, so when the liquid flowed through the rotating impeller, and the liquid stayed on the pressure surface of the blade. Under the action of the centrifugal force, the gas phase converged and fell off at the impeller outlet from the suction surface to the pressure surface, which led to the phase separation. When the inlet gas content α was 0.15, the gas phase occupied most of the space of the impeller channels, which may cause blockage in the flow channels. In this case, the distribution of liquid velocity streamlines on the cross-section of the impeller in Figure 7b showed that the gas phase accumulated to form a low-pressure area, which may cause a secondary flow and vortex.

**Figure 8:**
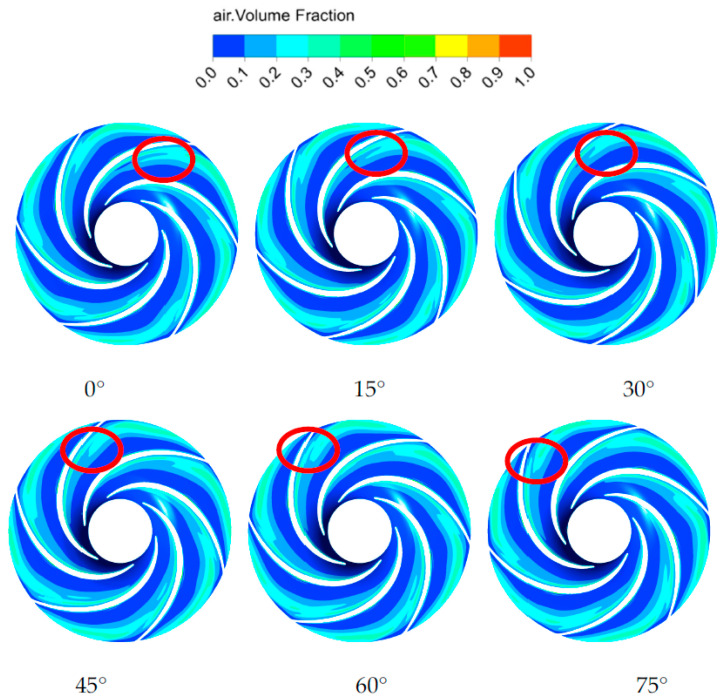
Transient distribution of the gas phase on the cross-section of the impeller under α = 0.1.

The transient flow field analysis was performed on the gas distribution in the impeller under an inlet gas content α of 0.1. Figure 8 shows the gas phase volume distribution on the cross-section under different impeller rotating angles. The distribution of the gas in the impeller channels varied with the rotation of the impeller. The gas phase was mainly gathered near the suction surface of the blades and gradually diffused along the impeller channels toward the outlet of the impeller. During the rotation of the impeller, the phenomenon of the gas accumulation and separation appeared along the flow direction. The separation phenomenon mainly occurred at the suction surface of the impeller blades adjacent to the outlet of the blades. Consequently, the gas in the impeller channels fell off at the outlet. The downstream part of the gas was separated from the impeller outlet and then entered the volute.

### 3.4. Effect of the Inlet Gas Content on the Impeller Radial Force Distribution

The radial force of a centrifugal pump with a volute is caused by the asymmetry of the volute structure on the circumference [30,31]. Under the condition of the gas-liquid two-phase flow, the internal flow field of the centrifugal pump is more complex than that under the single-phase flow, and the gas accumulation and separation may induce the phenomenon of a vortex and a secondary flow. In this study, the radial force from the impeller was monitored during the unsteady calculation. The calculation equation of the radial force was written as follows:

(1) $$F=\sqrt{F_{x}^{2}+F_{y}^{2}},$$

where *F*_x_ and *F*_y_ represent the components of radial force *F* in the *x* and *y* directions, respectively.

**Figure 9:**
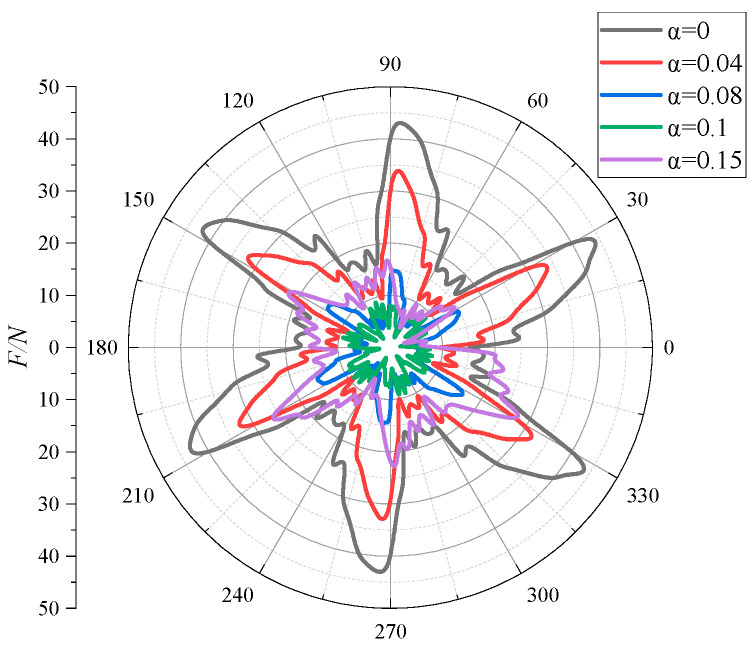
The radial force distributions of the impeller under different inlet gas fractions.

Figure 9 shows the radial force distribution of the impeller under different inlet gas contents at a rated flow. It can be seen from Figure 9 that the influence of the gas content change on the radial force was more obvious. The curve with an inlet gas content α of 0 was distributed in the outermost part. This curve shows a clear regularity, i.e., there are six distinct peaks in the curve, which are the same as the number of the blades of the impeller. It meant that when the inlet did not contain gas, the impeller was subjected to the greatest radial force and the curve fluctuation was strongly influenced by the number of blades. When the inlet gas content α increased from 0 to 0.1, the peak value of the radial force curve along the circumference gradually decreased. When the gas content reached 0.1, the curve fluctuation lost its original change law, and the number of peaks increased. The pattern of decreasing radial force due to the increasing gas content was similar to the research conclusions of Murakami et al. [32]. A small amount of gas in the impeller channels changed the flow velocity and direction of the original fluid, causing the change of radial force, so that the radial force on the impeller under gas-containing conditions was less than that under pure water conditions. For α = 0.15, the radial force curve fluctuated haphazardly and was no longer centrally symmetrical. The curve extreme large values in the range of 0–90° were smaller, compared with those in the other regions, but were still larger than the radial large values in the working condition with a gas content of 0.1. In combination with the above results, it was indicated that the magnitude of the radial force was nonpositively related to the gas content. The reason for this may be the change of the gas flow pattern in the impeller channels when the gas content reached a certain value [33]. The gas bubble flow aggregated into a slug flow, and the bubbles occupied most of the impeller channels. This resulted in the uneven distribution and the significant amplitude increase of the radial force on the impeller.

**Figure 10:**
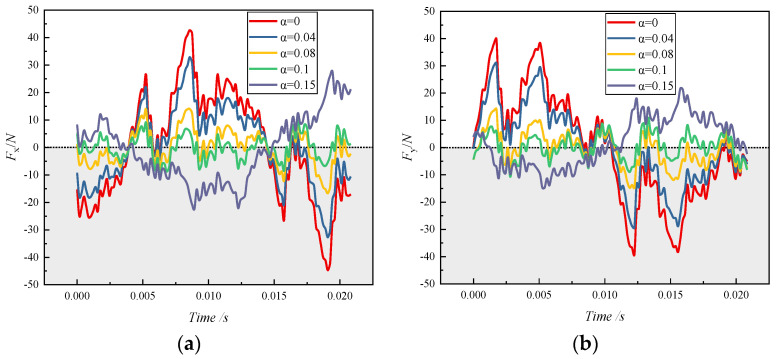
The time-domain distribution of the radial force component of the impeller under different inlet gas fractions: (**a**) *x* direction; (**b**) *y* direction.

The time-domain diagram shown in Figure 10 was the variation of the radial force component with times in the *x* and *y* directions. It can be seen that, as the inlet gas content increased, the radial force component in the *x* direction generally became smaller and the curve fluctuation patterns were similar. When the gas content reached 0.15, the law of the curve fluctuation was disrupted.

The changing pattern of radial force component curve in the *y* direction under each inlet gas condition was similar to that in the *x* direction, except that there was a phase difference between the curves with the same gas content in these two directions. With the variation of time, the radial force component fluctuated above and below the reference line 0. At gas contents of 0.08 and 0.1, the two component curves crossed the reference line several times, indicating that the direction of the radial force changed frequently under this gas-containing condition. It can be concluded that the gas-containing conditions caused a serious impact on the pump’s operating stability.

### 3.5. Effect of the Gas Content on the Pressure Pulsation

**Figure 11:**
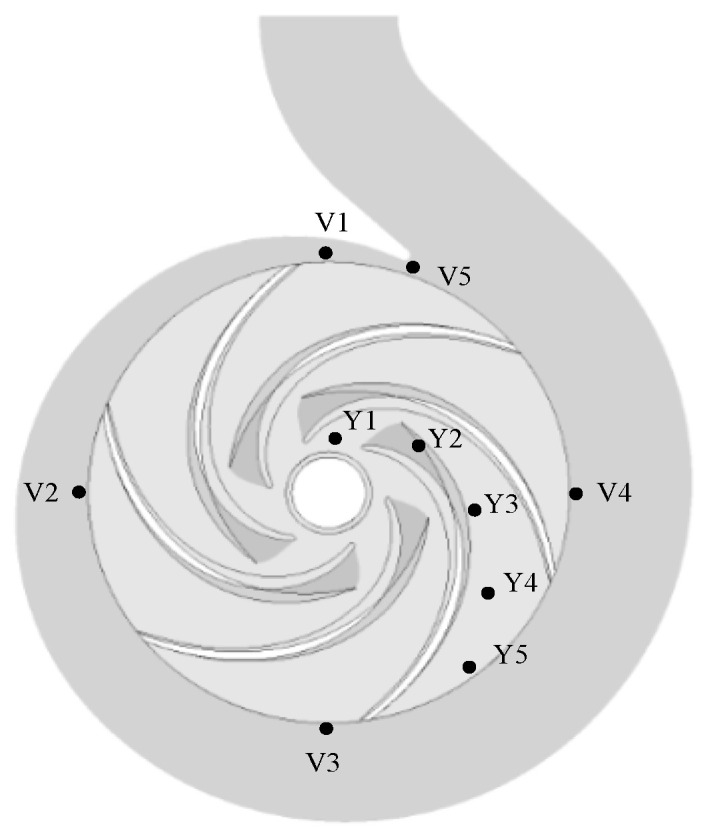
Positions of monitoring points.

To study the pressure fluctuation in the impeller channels and volute tongue under different gas content conditions, 10 monitoring points were set up in the flow channel, as shown in Figure 11.

The pressure data obtained from the numerical calculation of each monitoring point were treated with dimensionless processing [34,35]. The equation for the dimensionless processing of the pressure pulsation coefficient *C*_p_ was described as follows:

(2) $$C_{p}=\frac{p-\bar{p}}{\frac{1}{2}\rho u_{2}^{2}},$$

where *p* (unit: Pa) is the pressure at the monitoring point at different moments; $\bar{p}$ (unit: Pa) is the average pressure of the monitoring point; *ρ* (unit: kg/m^3^) is the fluid density; *u*_2_ (unit: m/s) is the impeller outlet circumferential velocity.

**Figure 12:**
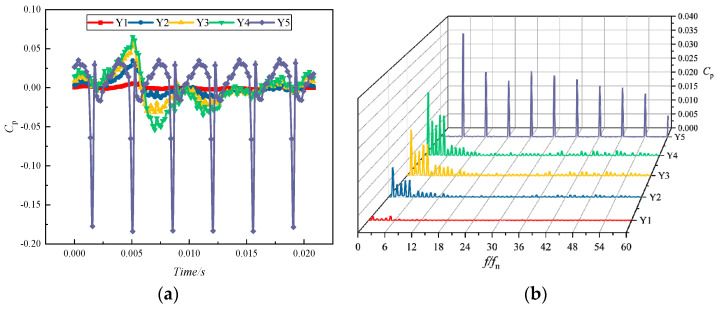
Pressure pulsation characteristics at each monitoring point in the impeller runner for α = 0: (**a**) time-domain distribution; (**b**) frequency-domain distribution.

Figure 12 reveals the time-domain and frequency-domain diagrams of the pressure pulsation at five monitoring points Y1–Y5 in the impeller channel under the conditions of a rated flow and an inlet gas content α of 0. From the time-domain distribution diagram, it is clearly seen that the pressure pulsation amplitude monitored at the monitoring point became more and more intense in the direction from the inlet to the outlet of the impeller channel. Meanwhile, the peaks and troughs tended to be more pronounced, and their quantities were the same as the number of the impeller blades Z, i.e., 6. Monitoring point Y1 was located at the impeller blade inlet, where the pressure pulsation was minimal and the pulsation curve was flat. Monitoring point Y5 was located at the outlet of the impeller, and the pressure pulsation at this monitoring point was the most obvious. This finding showed that the pressure signal near the blade outlet was larger in magnitude, i.e., the pressure pulsation signal was primarily generated at the blade outlet. The main reason is the dynamic and static interference between the impeller and the volute.

From the frequency-domain distribution diagram, the pulsation intensity of the pulsation source increased from the inlet to the outlet of the impeller. The main frequency of Y5 pulsation at the monitoring point appeared at one time of the blade-passing frequency (BPF). The pressure pulsation coefficient amplitude at each harmonic frequency decreased compared to the main frequency amplitude but differed from the rapid reduction in pressure pulsation amplitude at the other four monitoring sites [36]. Once again, the pulsation source was shown to arise primarily at the impeller outlet.

**Figure 13:**
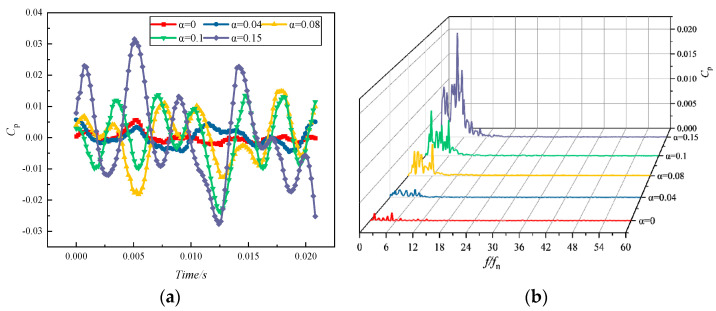
Pressure pulsation characteristics of monitoring point Y1 at different inlet gas fractions: (**a**) time-domain distribution; (**b**) frequency-domain distribution.

Figure 13 indicates the time- and frequency-domain diagrams of the pressure pulsation at monitoring point Y1 of the impeller blade inlet under the conditions of a rated flow and different inlet gas contents. As the inlet gas content increased, the amplitude of the pressure pulsation was enlarged, and the pulsation curve approximated a sine wave distribution. As the rate of the gas content increased, the pressure coefficient fluctuation curve became chaotic. This meant that the growth in gas content broke the stable operation of the pump, and the larger the gas content, the more turbulent the pulsating signal in the pump. It can be ascribed to the fact that the increase in the gas content in the impeller channel occupied more space, which caused the liquid to be squeezed out [37]. On this account, more vortices appeared. Subsequently, the liquid flow became more complicated, disrupting the original flow pattern and giving rise to unstable pump operation. The frequency-domain characteristics of monitoring point Y1 were similarly distributed under different imported gas content conditions. The main frequencies all occurred at BPF, and the pulsation amplitude at each harmonic frequency was almost zero. The intensity of the pulsation at one time frequency gradually became greater, as the inlet gas content rose. However, the principle of this enhancement is different from the principle, where the pulsation intensity became stronger as it got closer to the pulsation source. The rise in gas content did not produce a new pulsating signal other than the main-frequency and low-frequency signal enhancement.

**Figure 14:**
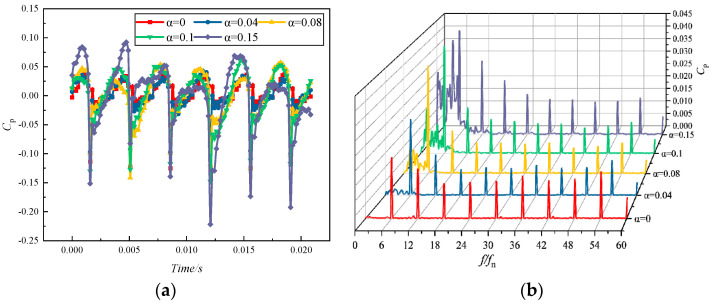
Pressure pulsation characteristics of monitoring point V5 at different inlet gas fractions: (**a**) time-domain distribution; (**b**) frequency-domain distribution.

Figure 14 illustrates the time-domain and frequency-domain diagrams of the pressure pulsation of the volute tongue separation at monitoring point V5 under the condition of a rated flow and different inlet gas contents. From the time-domain distribution, the pressure pulsation pattern at each monitoring point was approximately identical under different gas content conditions. The numbers of peaks and troughs were the same as the number of impeller blades. As the gas content increased, the pulsation amplitude interval was extended, and the pressure pulsation became more severe. Unlike the time-domain distribution of the monitoring site shown in Figure 13, the intensity of the pressure pulsation was greater. Meanwhile, there were significant troughs in the fluctuation curve. It can be attributed to the dynamic and static interferences between the rotating impeller and the stationary spiral casing at the tongue. The main frequency of the pressure pulsations with different gas contents was found at BPF and at its harmonics. The higher the gas content, the greater the amplitude of the pulsations below BPF. Thus, the presence of gas may cause the centrifugal pump to operate with more low-frequency pressure pulsations [38].

## 4. Conclusions

(1) Under the inlet gas condition, the energy performance curve of the model pump decreased with the growth of the gas content. The centrifugal pumps had a serious impact on the performance of eccentric conditions when pumping liquid-containing gas. The efficient operating range also showed a declining trend.

(2) Under the inlet gas condition, the gas phase was mainly distributed on the suction surface of the impeller blade. As the inlet gas content increased, the gas phase was gathered along the suction side at the impeller outlet, and the gas phase was gathered in the impeller runner to produce a low-pressure area. This induced a swirling, secondary flow and produced an airlock that blocked the flow channel.

(3) The change of the inlet gas content dramatically influenced the magnitude and the direction of the radial force on the impeller blades. The pure water condition had the largest radial force value. As the gas content increased, the radial force decreased accordingly. Under the condition of low gas content, the changing pattern was similar, and the number of the wave peaks was equal to the number of the impeller blades. The radial force fluctuated disorderly, after the gas content reached a certain value. The magnitude and the direction of the radial force fluctuated frequently, which had a negative effect on the operational stability of the pump.

(4) Under pure water conditions, the pressure pulsation intensity in the flow path of the impeller continuously increased along the flow direction. Under the condition of the gas-liquid two-phase flow, the pressure pulsation at the leading edge of the blade and the volute tongue became more and more intense with the increase of the inlet gas void fraction. The pulsation intensity of the pressure signal was enhanced by the increase in the gas content rate, which was different from the principle that the pulsation intensity becomes stronger when the monitoring point is closer to the pulsation source. However, the main frequency of the pressure pulsations occurred at BPF.

## Figures and Tables

**Table 1:** Main design parameters of a centrifugal pump.

Design Parameters	Value
Design head	35 m
Design volume flow rate	50 m^3^/h
Design speed	2850 r/min
Impeller inlet diameter	74 mm
Impeller outlet diameter	174 mm
Impeller outlet width	12 mm
Blade wrap angle	108°
Blade outlet angle	31°
Volute base circle diameter	184 mm
Inlet width of volute	20 mm
Number of blades	6

## References

[1] Sulzer Pumps (2010). Centrifugal Pump Handbook. https://www.sciencedirect.com/book/9780750686129/centrifugal-pump-handbook.

[2] Li W., Ji L., Shi W., Zhou L., Chang H., Agarwal R.K. (2020). Expansion of high efficiency region of wind energy centrifugal pump based on factorial experiment design and Computational Fluid Dynamics. Energies.

[3] Yang Y., Zhou L., Shi W., He Z., Han Y., Xiao Y. (2020). Interstage difference of pressure pulsation in a three-stage electrical submersible pump. J. Pet. Sci. Eng..

[4] Mauricio B., Alberto L., Jorge L., Verde W., Sassim N. (2020). Fault identification using a chain of decision trees in an electrical submersible pump operating in a liquid-gas flow. J. Pet. Sci. Eng..

[5] Bai L., Zhou L., Jiang X., Pang Q., Ye D. (2019). Vibration in a multistage centrifugal pump under varied conditions. Shock Vib..

[6] Peng G., Huang X., Zhou L., Zhou G., Zhou H. (2020). Solid-liquid two-phase flow and wear analysis in a large-scale centrifugal slurry pump. Eng. Fail. Anal..

[7] Qian J., Li X., Wu Z., Jin Z., Zhang J., Sunden B. (2019). Slug formation analysis of liquid-liquid two-phase flow in T-junction microchannels. J. Therm. Sci. Eng. Appl..

[8] Qian J., Hou C., Li X., Jin Z. (2020). Actuation Mechanism of Microvalves: A Review. Micromachines.

[9] Zhu J., Zhang J., Cao G., Zhao Q., Peng J., Zhu H., Zhang H.Q. (2019). Modeling flow pattern transitions in electrical submersible pump under gassy flow conditions. J. Pet. Sci. Eng..

[10] Yuan J., Deng F., Zhang K., Cui Q., Si Q. (2019). Status of research in internal flow in impeller pumps under gas-liquid two-phase conditions. J. Drain. Irrig. Mach. Eng..

[11] He D., Ge Z., Bai B., Guo P., Luo X. (2020). Gas-liquid two-phase performance of centrifugal pump under bubble inflow based on Computational Fluid Dynamics–Population Balance Model Coupling Model. ASME J. Fluids Eng..

[12] Ge Z., He D., Huang R., Zuo J., Luo X. (2020). Application of CFD-PBM coupling model for analysis of gas-liquid distribution characteristics in centrifugal pump. J. Pet. Sci. Eng..

[13] Sato S., Furukawa A., Takamatsu Y. (2008). Air-water two-phase flow performance of centrifugal pump impellers with various blade angles. Bull. JSME.

[14] Trupen P., Michael M., Dominique T. (2020). Investigations on the effect of tip clearance gap and inducer on the transport of air-water two-phase flow by centrifugal pumps. Chem. Eng. Sci..

[15] Poullikkas A. (2000). Two phase flow performance of nuclear reactor cooling pumps. Prog. Nucl. Energy.

[16] Matsushita N., Watanabe S., Okuma K., Hasui T., Furukawa A. Similarity law of air-water two-phase flow performance of centrifugal pump. Proceedings of the ASME/JSME 2007 5th Joint Fluids Engineering Conference.

[17] Pirouzpanah S., Gudigopuram S., Morrison G. (2016). Two-phase flow characterization in a split vane impeller Electrical Submersible Pump. J. Pet. Sci. Eng..

[18] Caridad J., Asuaje M., Kenyery F., Tremante A., Aguilon O. (2008). Characterization of a centrifugal pump impeller under two-phase flow conditions. J. Pet. Sci. Eng..

[19] Verde W., Biazussi J., Sassim N., Bannwart A. (2017). Experimental study of gas-liquid two-phase flow patterns within centrifugal pumps impellers. Exp. Therm. Fluid Sci..

[20] Cubas J., Stel H., Ofuchi E., Neto M., Morales R. (2020). Visualization of two-phase gas-liquid flow in a radial centrifugal pump with a vaned diffuser. J. Pet. Sci. Eng..

[21] Kosmowski I. Behaviour of centrifugal pump when conveying gas entrained liquids. Proceedings of the 7th Technical Conference of BPMA.

[22] Wang H., Long B., Wang C., Han C., Li L. (2020). Effects of the Impeller Blade with a Slot Structure on the Centrifugal Pump Performance. Energies.

[23] Zhou L., Deshpande K., Zhang X., Agarwal R. (2020). Process simulation of Chemical Looping Combustion using ASPEN Plus for a mixture of biomass and coal with various oxygen carriers. Energy.

[24] Zhang Y., Fernandez-Rodriguez E., Zheng J., Zheng Y., Zhang J., Gu H., Zang W., Lin X. (2020). A review on numerical development of tidal stream turbine performance and wake prediction. IEEE Access.

[25] Cheng H., Bai X., Long X., Ji B., Peng X., Farhat M. (2020). Large eddy simulation of the tip leakage cavitating flow with an insight on how cavitation influences vorticity and turbulence. Appl. Math. Model..

[26] Hou G., Taherian H., Li L., Fuse J., Moradi L. (2020). System performance analysis of a hybrid ground source heat pump with optimal control strategies based on numerical simulations. Geothermics.

[27] Wang C., Shi W., Wang X., Jiang X., Yang Y., Li W., Zhou L. (2017). Optimal design of multistage centrifugal pump based on the combined energy loss model and computational fluid dynamics. Appl. Energy.

[28] Zhou L., Wang W., Hang J., Shi W., Yan H., Zhu Y. (2020). Numerical investigation of a high-speed Electrical Submersible Pump with different end clearances. Water.

[29] Li W., Li E., Ji L., Zhou L., Shi W., Zhu Y. (2020). Mechanism and propagation characteristics of rotating stall in a mixed-flow pump. Renew. Energy.

[30] Dong L., Shang H., Zhao Y., Liu H., Dai C., Wang Y. (2019). Study on unstable characteristics of centrifugal pump under different cavitation stages. J. Therm. Sci..

[31] Tan L., Shi W., Zhang D., Zhang Y., Zhang W. (2017). Effects of blade outlet angle on the performance for single blade pumps. J. Drain. Irrig. Mach. Eng..

[32] Murakami M., Minemura K. (1974). Effects of entrained air on the performance of centrifugal pumps: 2nd report, effects of number of blades. Trans. Jpn. Soc. Mech. Eng..

[33] Zhou L., Han C., Bai L., Shi W., Agarwal R. (2020). Numerical and experimental study of multiphase transient core-annular flow patterns in a spouted bed. ASME J. Energy Resour. Technol..

[34] Yang J., Liu J., Liu X., Xie T. (2019). Numerical study of pressure pulsation of centrifugal pumps with the compressible mode. J. Therm. Sci..

[35] Wei H., Xuefeng L.I., Min S.U., Rennian L.I., Hao C. (2018). Pressure fluctuation of solid-liquid flow in stator and rotor cascades of pump as turbine. J. Drain. Irrig. Mach. Eng..

[36] Zhang Y., Zhang J., Lin X., Wang R., Zhang C., Zhao J. (2020). Experimental investigation into downstream field of a horizontal axis tidal stream turbine supported by a mono pile. Appl. Ocean Res..

[37] Jin Z., Qiu C., Jiang C., Wu J., Qian J. (2020). Effect of valve core shapes on cavitation flow through a sleeve regulating valve. J. Zhejiang Univ. Sci. A..

[38] Qian J., Li X., Wu Z., Jin Z., Sunden B. (2019). A comprehensive review on liquid-liquid two-phase flow in microchannel: Flow pattern and mass transfer. Microfluid. Nanofluid..

